# Plasmonic colloidosomes of black gold for solar energy harvesting and hotspots directed catalysis for CO_2_ to fuel conversion†
†Electronic supplementary information (ESI) available: Detailed experimental surface area data, low magnification STEM images, PXRD, details of thermal efficiency and Raman thermometry calculations, catalysis selectivity data and tomography videos. See DOI: 10.1039/c9sc02369k


**DOI:** 10.1039/c9sc02369k

**Published:** 2019-07-03

**Authors:** Mahak Dhiman, Ayan Maity, Anirban Das, Rajesh Belgamwar, Bhagyashree Chalke, Yeonhee Lee, Kyunjong Sim, Jwa-Min Nam, Vivek Polshettiwar

**Affiliations:** a Department of Chemical Sciences , Tata Institute of Fundamental Research (TIFR) , Mumbai , India . Email: vivekpol@tifr.res.in; b Department of Condensed Matter Physics and Materials Science , Tata Institute of Fundamental Research (TIFR) , Mumbai , India; c Department of Chemistry , Seoul National University , Seoul , South Korea

## Abstract

In this work, we showed the tuning of the catalytic behavior of dendritic plasmonic colloidosomes (DPCs) by plasmonic hotspots. A cycle-by-cycle solution-phase synthetic protocol yielded high-surface-area DPCs by controlled nucleation–growth of gold nanoparticles. These DPCs, which had varying interparticle distances and particle-size distribution, absorb light over the entire visible region as well as in the near-infrared region of the solar spectrum, transforming gold into black gold. They produced intense hotspots of localized electric fields as well as heat, which were quantified and visualized by Raman thermometry and electron energy loss spectroscopy plasmon mapping. These DPCs can be effectively utilized for the oxidation reaction of cinnamyl alcohol using pure oxygen as the oxidant, hydrosilylation of aldehydes, temperature jump assisted protein unfolding and purification of seawater to drinkable water *via* steam generation. Black gold DPCs also convert CO_2_ to methane (fuel) at atmospheric pressure and temperature, using solar energy.

## Abstract 2

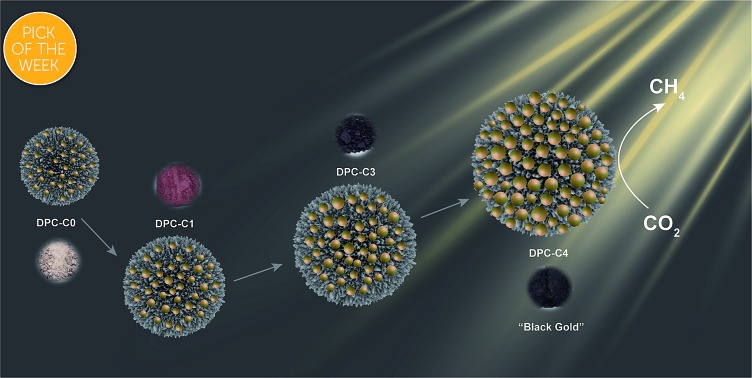
Plasmonic black gold converts CO_2_ to methane using solar energy.

## 1. Introduction

Localized surface plasmon resonance (LSPR), which provides a combination of localized heat and electric fields, was used for the development of efficient catalytic and solar energy harvesting processes.^1^–^6^ Recently Jain *et al.* showed the unique application of plasmonic nanoparticles for CO_2_ conversion by the concept of electron injection from the Fermi level of excited gold (Au) nanoparticles (NPs) to the reactant molecules.^7^–^9^ Camargo's group, on the other hand, showed the tuning of activity and selectivity of plasmonic gold nanoparticles for organic transformations by the concept of hybrid materials and metal–support interactions.^10^–^12^

Tuning the LSPR of the material to optimize the electromagnetic (EM) and thermal hotspots has scientific and technological advantages. Plasmonic coupling, a near-field coupling of the dipoles in the presence of light, can also be tuned by modifying the gap/distance between NPs.^13^ Unfortunately tuning of interparticle distances on the support is a significant challenge and was only achieved by instrumental techniques like lithography, atomic layer deposition, *etc.*^14^,^15^ These techniques cannot be used to design heterogeneous catalysts, which require a high loading of Au and high surface area (porosity) for improved light harvesting and efficient mass transport (diffusion). Colloidosomes can provide broadband plasmonic materials, which were effectively used for surface-enhanced Raman spectroscopy (SERS) though they exhibit low surface area.^16^,^17^

In this work, we developed a novel solution phase synthetic protocol for dendritic plasmonic colloidosomes (DPCs) by controlled nucleation–growth of Au NPs onto high-surface-area dendritic fibrous nanosilica^18^,^19^ with varying interparticle distances and particle size distributions. We hypothesized that the distribution of particle sizes and the plasmonic coupling between Au NPs would lead to optimum generation of heat *via* “thermal hotspots” and electric fields *via* “EM hotspots”. The main goal of this work was to establish whether tuning of these hotspots allows tuning of the solar energy harvesting and catalytic activity of these plasmonic colloidosomes.

## 2. Results and discussion

### 2.1 Synthesis of dendritic plasmonic colloidosomes using a cycle-by-cycle growth approach

**Fig. 1:**
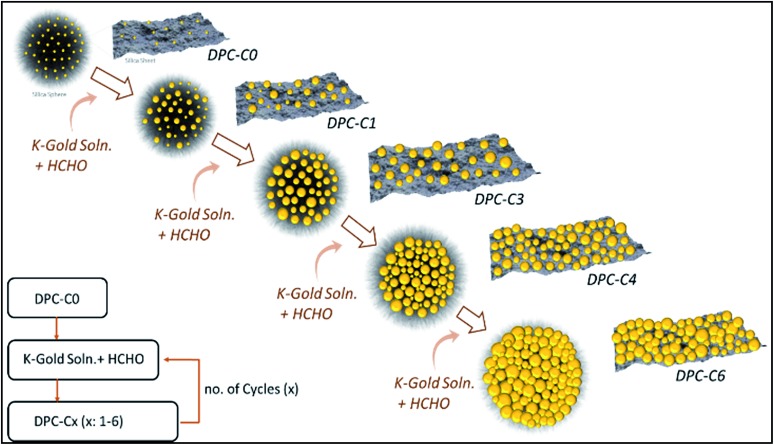
Cycle-by-cycle growth approach for dendritic plasmonic colloidosomes with varying Au particle sizes and interparticle distances.

The design and synthesis of DPCs that satisfy the above objectives was challenging, as it required high loading of gold with control over both the Au NP size and the distance between them on the support surface without reducing the surface area and porosity. We achieved this by using dendritic fibrous nanosilica, whose fibers (sheets) were used as the deposition site for the Au NPs, and then varying the distances between the Au NPs by using a cycle-by-cycle growth approach *via* a controlled nucleation–growth synthetic protocol (Fig. 1).

Amine functionalized nanosilica was decorated with nearly-monodisperse Au NPs (2–3 nm in size) by reduction with sodium borohydride (NaBH_4_). To vary the distance between the Au NPs, we decided to increase the size of the Au NP seeds, as with the increase in the size of Au NPs, the distance between them will be reduced (Fig. 1). This was attempted by selective growth of the existing Au NPs in DPC-C0 without forming new seeds, by avoiding the super-saturation regime of Au^0^ species (required for the nucleation and formation of new Au seeds). This was achieved by converting the Au precursor to a less reactive K-gold solution and also by replacing NaBH_4_ with the weak reducing agent formaldehyde (HCHO). This allowed for the slow formation of Au^0^ species, avoiding super-saturation, and hence, only the growth of the existing Au seeds in DPC-C0 took place, yielding DPC-C1. By repeating this growth cycle, DPC-C3, DPC-C4, and DPC-C6 were synthesized, with reduced interparticle distances (Fig. 1). Complete prevention of nucleation was not possible, and a few nuclei formed during the growth cycles, leading to heterogeneity in the Au NP sizes.

**Fig. 2:**
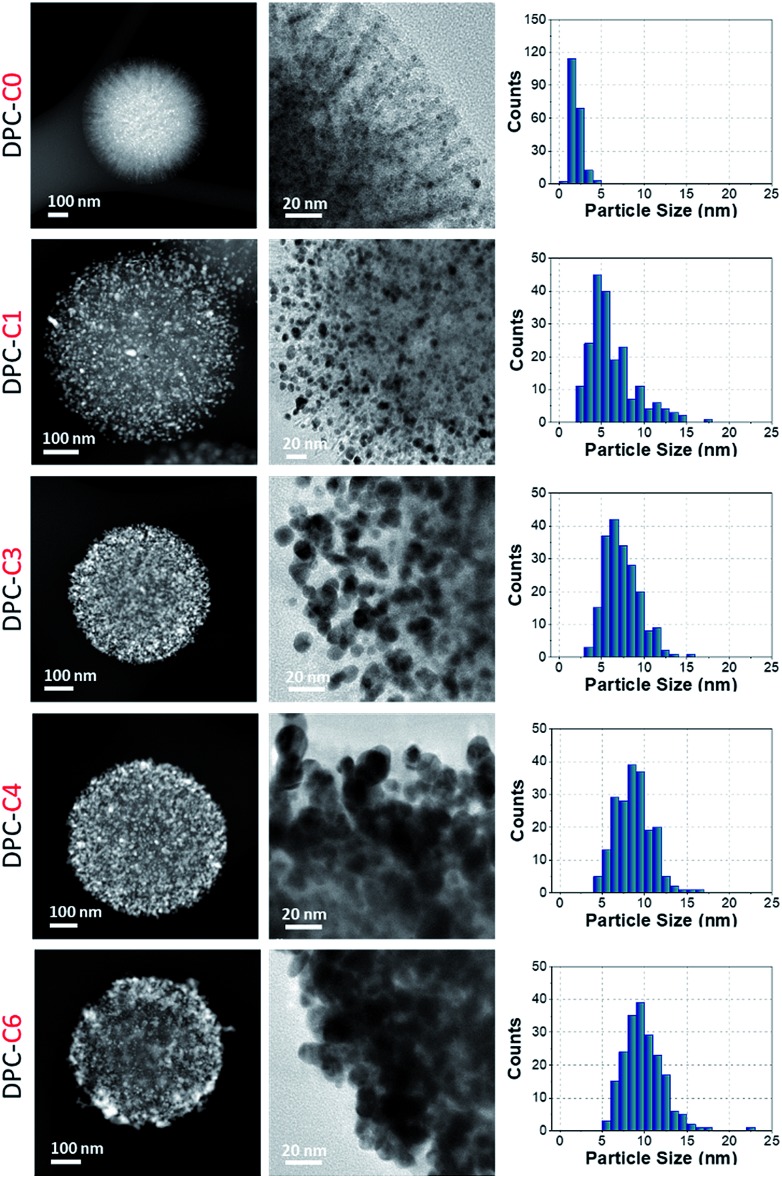
TEM images and particle size distributions of DPC-C*x*. These are representative images presenting statistically significant numbers of particles.

DPCs were characterized by scanning transmission electron microscopy (STEM) for the size and distribution of Au NPs (Fig. 2). Energy dispersive X-ray spectroscopy (EDS) was used to estimate the Au loading (Table S1†) and to visualize the spatial distribution of Au on silica spheres (Fig. S1†). STEM images of DPC-C0 indicate the formation of Au NPs, with average nanoparticle sizes of ∼3 nm, while DPC-C1 had a Au loading of 22 wt% and average particle size of ∼5 nm, which were uniformly distributed over the entire silica sphere (Fig. 2 and S1†). DPC-C3 and C4 had a 48 and a 55 wt% Au loading, with a particle size of ∼7.4 and 8.6 nm, respectively (Fig. 2 and S2†). In DPC-C6 with 68 wt% Au, most of the Au NPs were connected and the silica spheres seemed to be completely coated with Au NPs (Fig. 2 and S2†).

3D tomography study of DPC-C3, -C4 and -C6 (ESI Videos†) showed that there was heterogeneity in the particle sizes, with smaller particles inside and larger particles at the periphery of the silica spheres. Owing to the heterogeneity in the particle sizes and their distribution as well as the complex 3D fibrous structure of the silica spheres, calculation of the interparticle distances was not possible. However, since the particle sizes increased with the increasing number of growth cycles and increasing crowding of Au NPs on the silica spheres was observed in TEM images (Fig. 2), we statistically assumed that the average distances between the Au NPs decreased with an increasing number of growth cycles.

### 2.2 Tuning of the LSPR by optimizing the interparticle distances between Au NPs

**Fig. 3:**
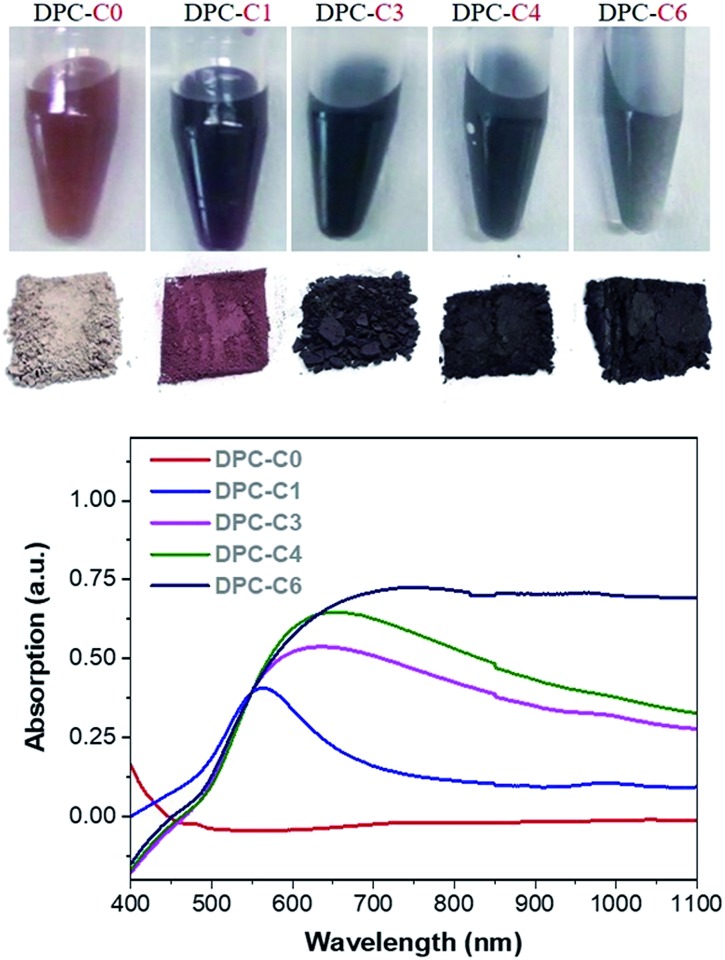
(Top) The color of DPC-C*x* dispersed in water and powder, and (bottom) absorption spectrum of DPC-C*x* with various numbers of growth cycles.

The physical texture and color of the DPC-C*x* materials indicated the systematic transformation of pale yellow to black gold from DPC-C0 to C6 (Fig. 3, top). The progressive nature of light absorption by these DPCs, with a gradual increase over the entire visible to NIR region, with an increasing number of gold cycles on the silica spheres (Fig. 3 and S3a†) indicated the roles of plasmonic coupling and varying particle-size distribution. Notably, these materials started absorbing light over the entire visible and near-infrared regions of the solar spectrum (Fig. S3a†). Nitrogen adsorption–desorption studies indicated that even after high gold loading, the DPCs were able to maintain good porosity (Fig. S3b and Table S1†). Powder X-ray diffraction analysis indicated the formation of crystalline Au NPs with peaks at 38°, 44°, 65° and 77° corresponding to the (111), (200), (220) and (311) planes of Au (Fig. S4†).

### 2.3 Solar energy harvesting using DPC nanoheaters

**Fig. 4:**
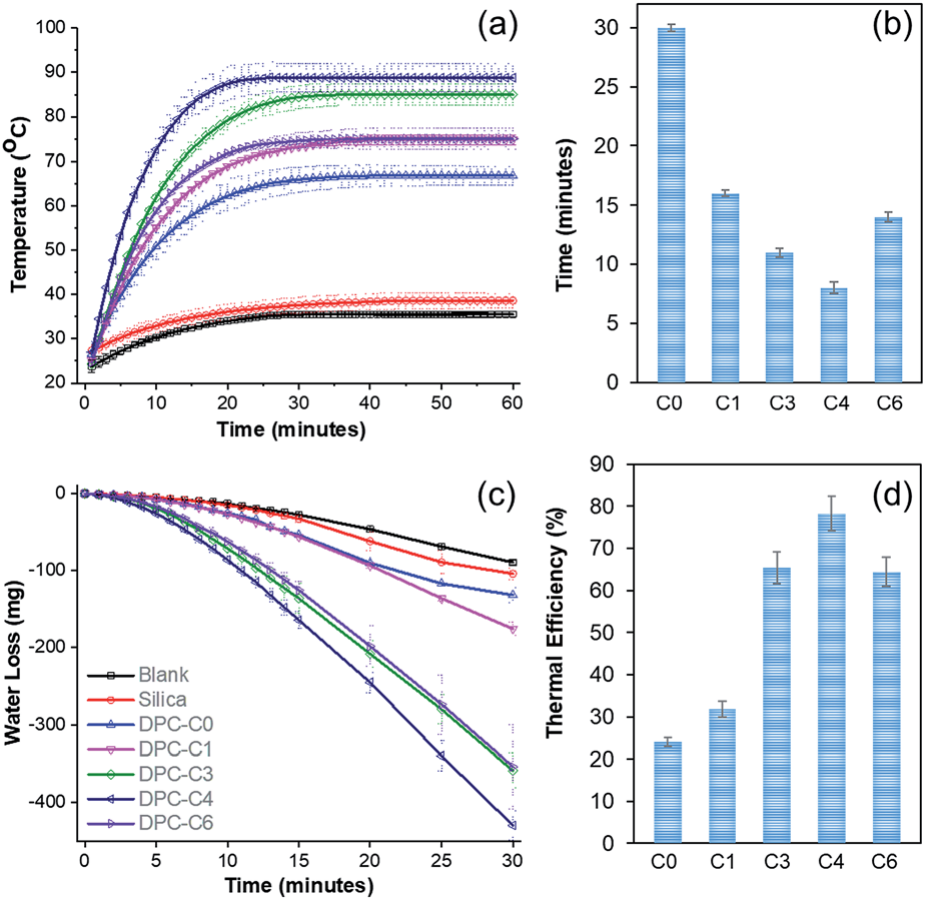
(a) Temperature of water with time, (b) kinetics of heating (time required to reach 65 °C), (c) steam generation (loss of water) over time, and (d) thermal efficiency of steam generation by plasmonic DPC-C*x*, using visible to NIR light (400 to 1100 nm wavelength).

To study the solar energy harvesting ability of these DPCs and also to get an insight about the thermal hotspots, we dispersed DPC-C*x* materials in water and exposed them to light (400 to 1100 nm) for 1 h and the temperature of the solution was measured over time (Fig. 4a). The temperature of the solution with pure silica spheres rose to 38 °C, while DPC-C0 and DPC-C1 heated the water to 67 and 75 °C, respectively. DPC-C3 and DPC-C4 further heated the water to 85 °C and 88 °C, respectively. The maximum increase in temperature by DPC-C4 was attributed to the heterogeneity of the particle sizes as well as optimum interparticle plasmonic coupling between Au NPs, resulting in a maximum number of thermal hotspots.

Interestingly, DPC-C6, despite having the highest Au loading, heated water only to 75 °C (Fig. 4a), as most of its Au NPs were connected, which drastically reduced the number of gaps present between the particles, *i.e.*, reducing the number of hotspots. In terms of the kinetics of water heating (Fig. 4b), DPC-C4 heated water to 65 °C in just 8 min, while the other samples needed more time, ranging from 13 to 30 min. This observation further confirmed the presence of a greater number of hotspots in DPC-C4. DPC-C4 was stable and recyclable over several heating–cooling cycles (Fig. S5a†). When water was replaced by other solvents, no significant difference in the heating behavior of DPC-C4 was observed (Fig. S5b†).

Notably, DPC-C4 was also used as a nano-heater to convert seawater into drinkable water, with an excellent efficiency (Fig. S5c†). These results indicate the potential application of DPCs in the purification of seawater to potable water *via* steam generation using solar energy under atmospheric reaction conditions. The steam generation experiment confirmed that the maximum number of hotspots was present in the DPC-C4 sample (Fig. 4c and d). The thermal efficiency of the steam generation process (Table S2†)^21^ was also found to be best for DPC-C4, which exhibited a 78% efficiency for converting water into steam (Fig. 4d).

**Fig. 5:**
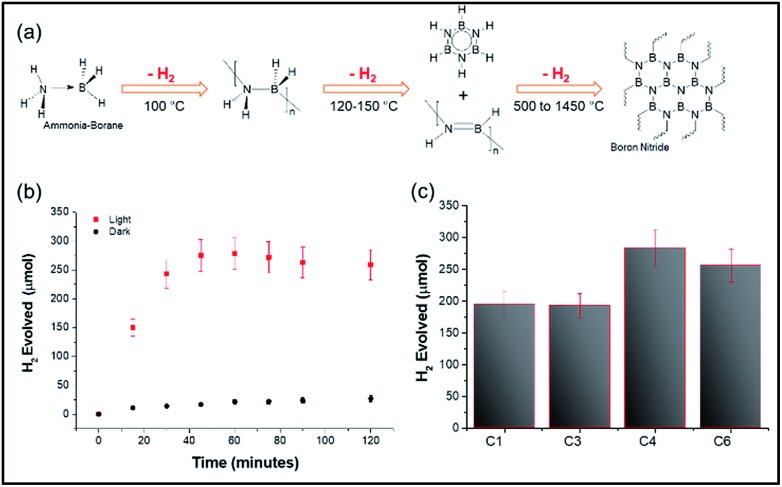
(a) Schematic of the temperature-dependent decomposition of the ammonia borane complex, ABC (100 μmol) decomposition by plasmonic (b) DPC-C4 under light and dark conditions and (c) DPC-C*x*, using visible to NIR light (400 to 1100 nm wavelength).

The above heating studies however did not provide information about absolute localized temperatures; hence we chose the decomposition of the ammonia borane complex (ABC) as a temperature-probe reaction.^20^ The ABC thermally decomposes in three consecutive steps, giving three equivalents of hydrogen, at approximately 110 °C, 150 °C, and <500 °C, with 1 molar equivalent of hydrogen released by each step (Fig. 5a). Thus, moles of hydrogen evolved could be directly related to the surface temperature of the materials. DPC-C4 produced 284 μmol of hydrogen from 100 μmol of ammonia borane complex after 2 h of light exposure, in line with the theoretical 300 μmol of H_2_. Notably, the trend of the number of growth cycles *vs.* hotspot formation was also followed here, with DPC-C4 showing the maximum moles of hydrogen evolved compared to that of C3 and C6 (Fig. 5b and c). However, upon exposure to light for 2 h, the temperature of the solution containing DPC-C4 rose to 74 °C, and when the dark reaction was carried out at this temperature, 200 μmol of H_2_ was evolved (Fig. S6†). These results indicate that some of the Au NPs in DPC-C*x* also behaved as catalytic sites and hence, ABC decomposition was not purely thermal (Fig. 5c).

### 2.4 Raman thermometry using Stokes and anti-Stokes SERS

**Fig. 6:**
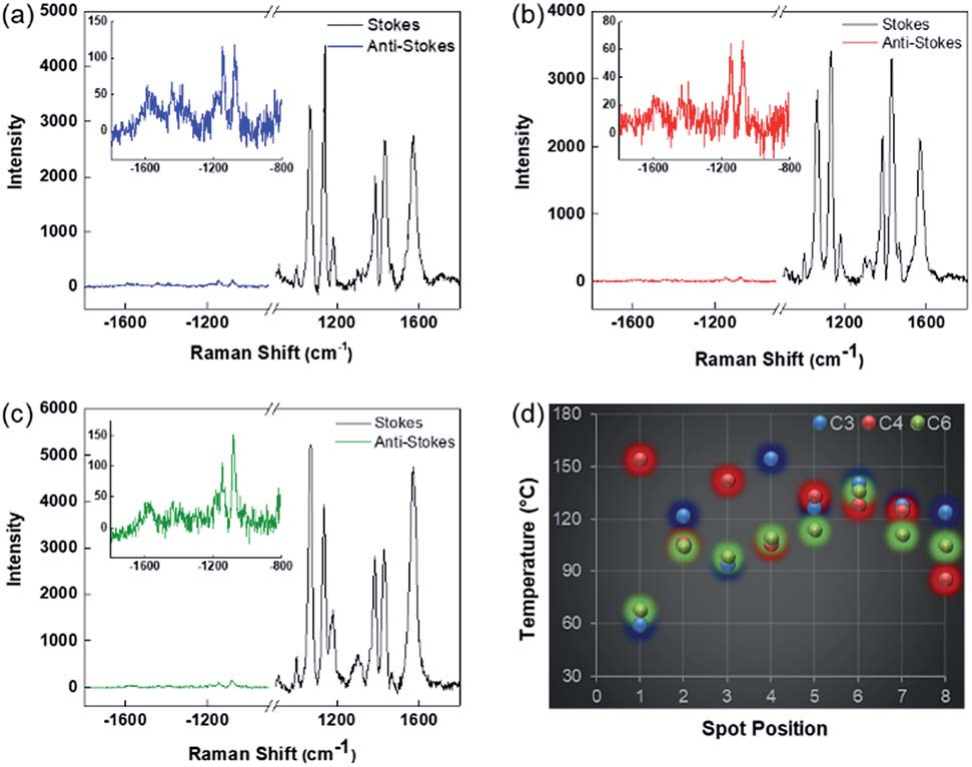
Representative Stokes and anti-Stokes SERS of 4-ATP on (a) DPC-C3, (b) DPC-C4, and (c) DPC-C6 (insets show enlarged anti-Stokes scattering) and (d) localized temperatures at different points on the aggregates of DPC-C*x*.

To find accurate local-temperature information, SERS-based Raman thermometry was used.^22^ During SERS, Stokes and anti-Stokes scattering signals were collected (Fig. 6), as the ratio of their intensities is proportional to the populations of their respective vibrational states given by the Boltzmann distribution. To minimize the effects of heterogeneity in the Au loading, data from eight different points for each sample were collected. Local temperatures were estimated using the following equation (the detailed calculation is given in Table S3†).
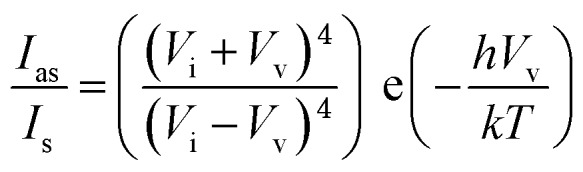
where *I*_as_ is the anti-Stokes signal intensity, *I*_s_ is the Stokes signal intensity, *V*_i_ is the laser frequency (in cm^–1^), *V*_v_ is the vibrational mode frequency (in cm^–1^), *h* is Planck's constant = 6.626176 × 10^–34^ J s, *k* is Boltzmann's constant = 1.3807 × 10 ^–23^ J K^–1^, and *T* is the temperature (in K).

The results clearly indicated that there is a significant difference between the bulk temperature of a solution of DPC-C*x*, which was in the range of 60 to 90 °C, and the local temperature (60 to 155 °C) at the surface of the Au NPs due to the loss of heat during convection from the Au NP surface to the surrounding environment. Notably, DPC-C3 and DPC-C4 have higher surface temperatures than DPC-C6 (Fig. 6d), confirming previous experimental (heat and steam) observations of the presence of more and energetic hotspots in C3 and C4 and its dependence on the inter-particle plasmonic coupling.

**Fig. 7:**
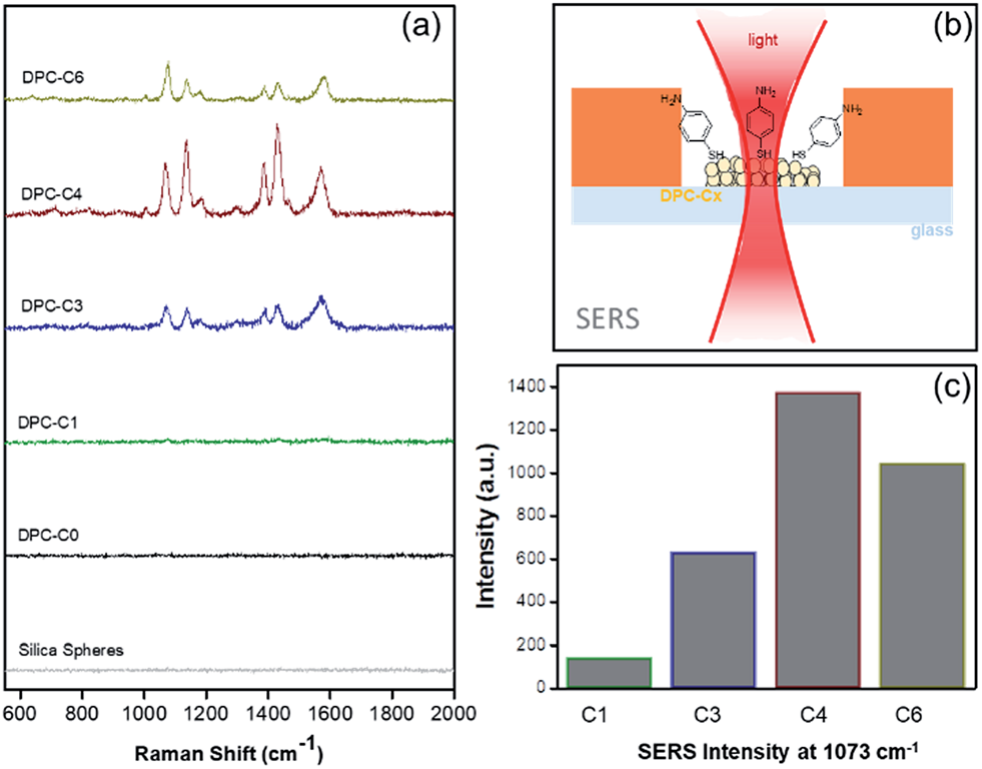
(a) SERS of 4-aminothiophenol on plasmonic DPC-C*x*, (b) SERS set-up, and (c) SERS intensities at 1073 cm^–1^ using DPC-C*x*.

The above experiments only probed “thermal hotspots” and to probe the “electromagnetic (EM) hotspots” of the synthesized materials, we performed surface-enhanced Raman spectroscopy,^23^ using 4-aminothiophenol (4-ATP) as the probe molecule using a 633 nm wavelength laser (Fig. 7 and Table S4†). Notably, as the gold NP crowding increased, there was a significant increase in the Raman peak intensity and was found to be at the maximum in DPC-C4, based on the intensity of the band at *ca.* 1073 cm^–1^ for C–S stretching (Fig. 7c). This was attributed to the optimum interparticle distances in DPC-C4, which led to the formation of a maximum number of EM hotspots. DPC-C6 with the highest Au loading and light absorption still showed lower SERS intensity than DPC-C3 and C4 (Fig. 7c), due to reduction in nanogaps between Au NPs.

### 2.5 Visualizing the hotspots by EELS plasmonic mapping

**Fig. 8:**
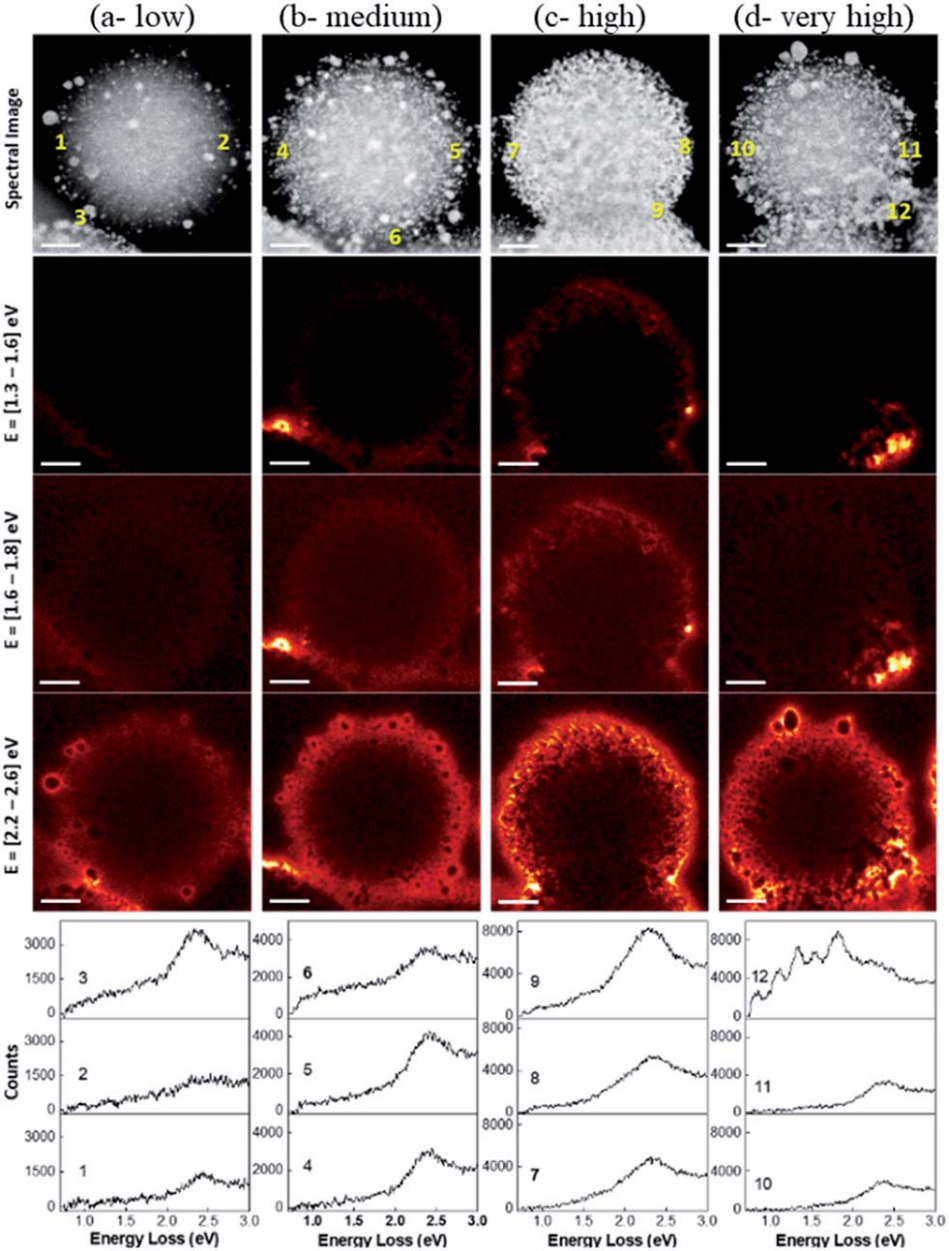
STEM–EELS plasmon mapping and EELS spectra at various spots in the DPC-C4. The brightness of the orange color indicates signal intensity.

The spatial distribution of localized surface plasmon modes was visualized by scanning transmission electron microscopy–electron energy loss spectroscopy (STEM–EELS) plasmon mapping.^24^ Heterogeneity in the Au loading allowed us to study the effects of Au–Au particle distances and particle size distributions on plasmonic hotspot formation by analyzing only one sample but providing information on DPC-C1, C3, C4 and C6 samples, which contained spheres with similar Au NPs crowding. The plasmonic behavior of the DPC materials is highlighted in three main electron-loss regions: 1.3–1.6 eV, 1.6–1.8 eV, and 2.2–2.6 eV. The EELS intensity in the different maps was found to be much higher in the energy range of 2.2–2.6 eV than in the energy ranges of 1.6–1.8 eV and 1.3–1.6 eV (Fig. 8). This directly correlates with the absorption spectrum (Fig. 3), where the maximum absorption for DPC-C3, C4, and C6 occurs at 540–560 nm (∼2.2 to 2.3 eV). The presence of modes below 2.4 eV confirms the longitudinal dipolar coupling of the Au nanoparticles, which can be related to the interparticle distance between Au NPs of the different samples.

The increase in crowding as well as the heterogeneity of the Au NPs (reduced interparticle distances) caused more intense hotspot formation, as shown in the 2nd column of Fig. 8. With a further increase in crowding, in the case of Fig. 8c spheres, hotspots became more intense and could even be seen at a lower energy loss of 1.3 to 1.6 eV. Notably, with the further increase in Au-crowding in Fig. 8d, there was a decrease in hotspot intensities (Fig. 8, 4th column), as in these spheres, and most Au NPs are connected to each other reducing the number of gaps between them (Fig. 8d).

## 2.6 Plasmonic hotspots directed catalysis and mechanistic study

To study the effect of plasmonic hotspots on catalysis and gain mechanistic understanding, we conducted four different studies using these DPCs, (i) oxidation reaction of cinnamyl alcohol using pure oxygen as the oxidant, (ii) CO_2_ to methane conversion at atmospheric pressure and temperature using water as the hydrogen source, (iii) protein denaturation studies, and (iv) hydrosilylation of aldehydes.

**Fig. 9:**
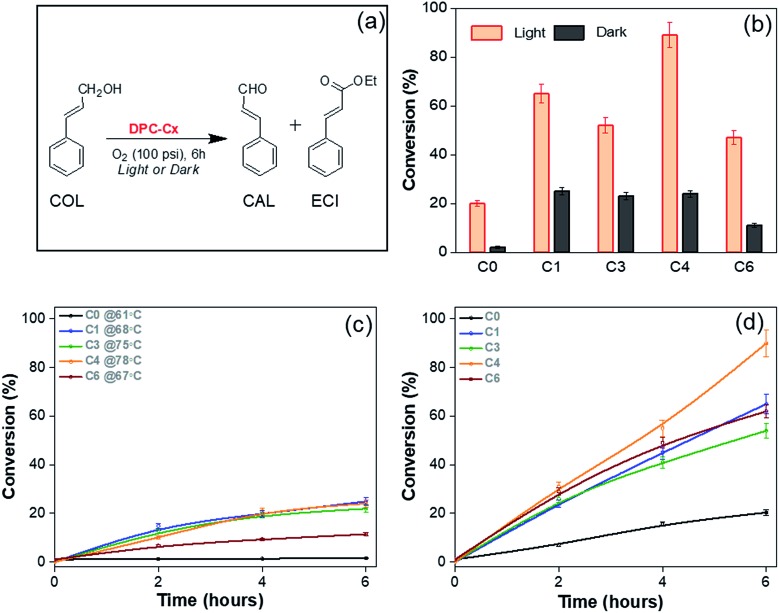
Cinnamyl alcohol oxidation by plasmonic DPC-C*x*, (a) reaction scheme, (b) conversion under light and dark conditions; reaction kinetics in the (c) dark at the respective temperatures and under (d) light. The amount of Au was the same for all the reactions. The light used was 400 nm–1100 nm with 1 W cm^–2^ power.

We first chose cinnamyl alcohol (COL) oxidation to cinnamaldehyde (CAL) as a test reaction (Fig. 9). For the oxidation of COL to CAL (Fig. 9a), the best reaction conditions were found to be DPC-C4 (10 mg), COL (0.1 mmol), O_2_ (100 psi), ethanol (5 mL) and reaction time (6 h) (Table S5†). As previously realized, when the light is incident on the DPC-C*x* samples, the temperature of the solution rises, and hence dark reactions were carried out under externally heated conditions, using DPC-C0, C1, C3, C4, and C6 at 61, 68, 75, 78 and 67 °C, respectively. Furthermore, since DPC-C*x* has different weight percentages of gold loading, the reaction was carried out using fixed moles of Au. The negligible conversion was observed in the blank and with pure silica spheres, while all of the DPC-C*x* catalysts showed high catalytic activity in the presence of light as compared to that in the dark (Fig. 9b). A maximum conversion of approximately 90% was obtained under light irradiation in the case of C4, with good selectivity (Fig. S7†). DPC-C4 also showed a better reaction rate than C1, C3, and C6 (Fig. 9d). Under dark conditions at 78 °C, C4 only resulted in a 24% conversion (Fig. 9c). Similarly, DPC-C3 and C6 resulted in higher conversions of COL to CAL, *i.e.*, 52% and 47% under light, with a comparable selectivity of 85 and 75% compared with those obtained under dark conditions (Fig. 9 and S8†).

Higher activity under light can be explained based on the varying numbers of EM and thermal hotspots. At the microscopic level, when the COL is positioned within the nano-gap of DPC-C*x*, the COL molecule experiences dramatically amplified EM fields. This facilitates the strong coupling between the plasmons of DPC-C*x* and COL molecules, polarizing the COL molecules and weakening the bonds, which in turn enhanced the conversion as well as the kinetics of COL to CAL. Hence, more hotspots resulted in more polarization (EM hotspots) and localized thermal energy.

**Fig. 10:**
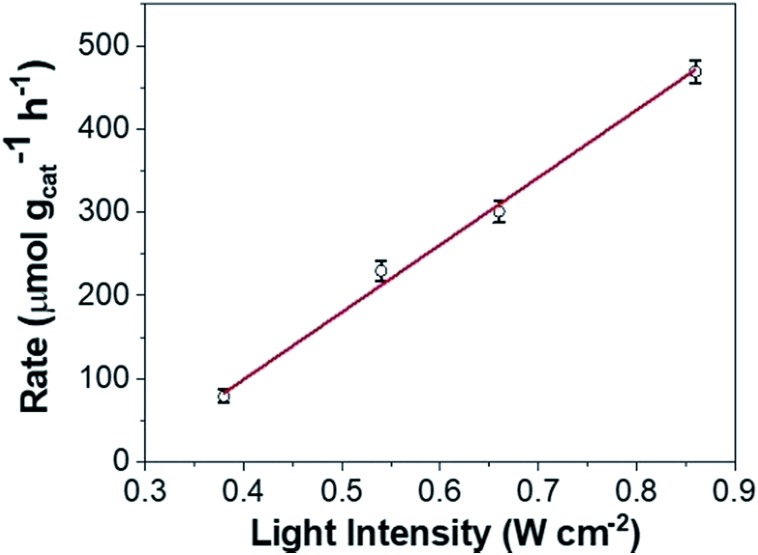
Intensity-dependent photocatalytic rate of COL to CAL using DPC-C4.

However, loss of SPR coherence in DPC-C*x* can also result in the opening of non-radiative decay pathways like Landau damping which can activate reactants by hot electron injection. Fig. 10 and S9† show the photocatalytic rate as a function of light intensity. The linear relationship between the photocatalytic rate and the light intensity is a known signature of electron mediated chemical reactions.^25^ This indicates that energetic electrons increase the overall reaction rate through an electron-assisted O_2_-dissociation process, *via* the transfer of an energetic electron from gold to the antibonding O–O 2p*-state of molecular O_2_ adsorbed on the gold surface. The use of an insulating silica support avoided any back-injection of these hot-electrons, further improving the probability transfer to an oxygen molecule.

Also, once hot electrons have undergone the fast electron–electron and electron–phonon scattering processes (10 fs–10 ps) to reach thermalization, the absorbed energy is transferred to the localized environment. Since COL oxidation is intrinsically an endothermic reaction, any amount of localized heat will bring about the favorable condition for the reaction to proceed. Thus, the formed highly localized thermal hotspots on the nanoparticles are an excellent source of localized heat that gets transferred to COL molecules in their proximity. These results indicate that the combination of energetic electrons, as well as hotspots (EM and thermal), works cooperatively and is responsible for the enhanced photoactivity. Since DPC-C4 possesses optimized hot-spots, it showed the best catalytic activity, over other DPCs.

**Fig. 11:**
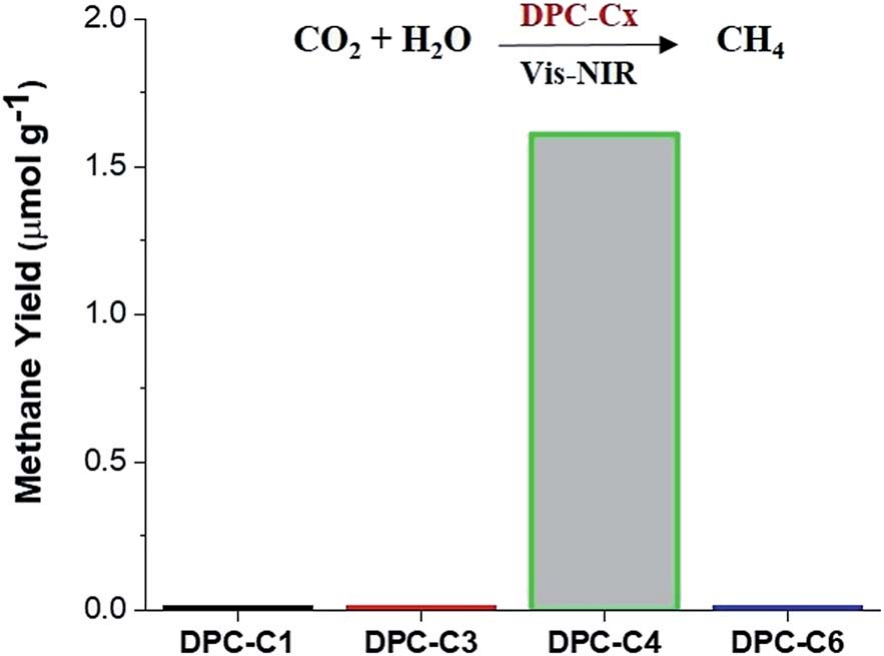
Photocatalytic CO_2_ conversion to methane using DPC-C1, DPC-C3, DPC-C4, and DPC-C6 as catalysts under light (400 to 1100 nm). No conversion in the dark or in the absence of CO_2_. Micro-GC with TCD sensitivity >10 ppm. Error in methane yield for DPC-C4: ±0.51.

To unequivocally study the effect of “hot-electron” transfer, without the participation of the thermal effect, we evaluated these DPC-C*x* for CO_2_ methanation reaction at atmospheric pressure and temperature (Fig. 11). Notably, only DPC-C4 catalyzed the methane production, while all other DPC-C*x* showed no methane or CO formation. It is known that for CO_2_ methanation, the rate-limiting step is electron injection from plasmonic Au to CO_2_ molecules.^8^ These results further confirm the role of energetic electron transfer and also indicate the potential to develop “hotspot” mediated CO_2_ conversion protocols.

**Fig. 12:**
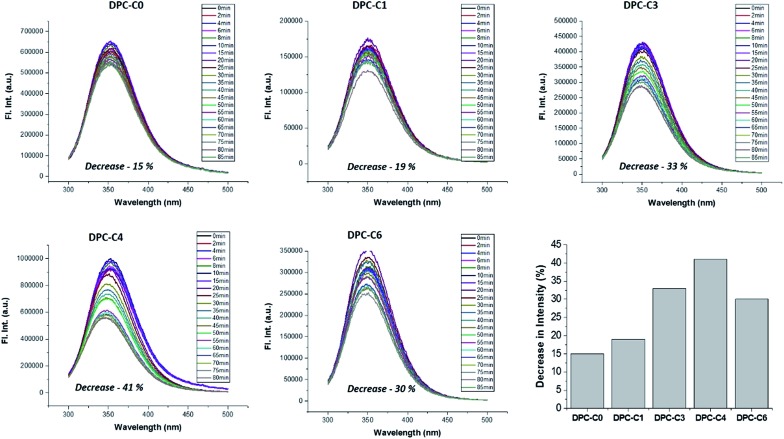
Steady-state fluorescence of 3.5 mL BSA solution (10 μM prepared in PBS buffer of 0.1 mM) in the presence of DPC-C0, DPC-C1, DPC-C3 and DPC-C6 in light (400 to 1100 nm). Excited at 280 nm and emission recorded from 300–500 nm.

On the other hand, to explicitly study the role of only “thermal hotspots”, we studied fundamental processes of protein folding and unfolding. Their folded form can be unfolded by a temperature jump, which can be monitored using fluorescence spectroscopy. This is purely a thermal effect and hence distinguishes the effect of thermal and electromagnetic plasmonic hotspots. We carried out a protein denaturation study of Bovine Serum Albumin (BSA) protein using DPC-C0, DPC-C1, DPC-C3, and DPC-C6 in the presence of light (400 to 1100 nm). The fluorescence behavior of two tryptophans (Trp-134 and Trp-213) in BSA was investigated by exciting the protein at 280 nm and recording the emission from 300–500 nm. The emission peak maxima for BSA (at 352 nm), on light illumination, due to the thermal effects of plasmonic DPC-C*x*, systematically decreased with time, indicating a global structural change (Fig. 12). A maximum of 41% decrease in peak intensity was observed for DPC-C4, followed by 33% for DPC-C3, 30% for DPC-C6, 19% for DPC-C1 and only 15% for DPC-C0. The relationship between the extent of decrease in fluorescence intensity and concentration of thermal hotspots again confirms the presence of more thermal hot-spots in DPC-C4. Using this approach protein denaturation can be performed at faster time scales by simply exposing the samples to the light, as compared to conventional thermal heating and can be potentially used in biomedical applications.

**Fig. 13:**
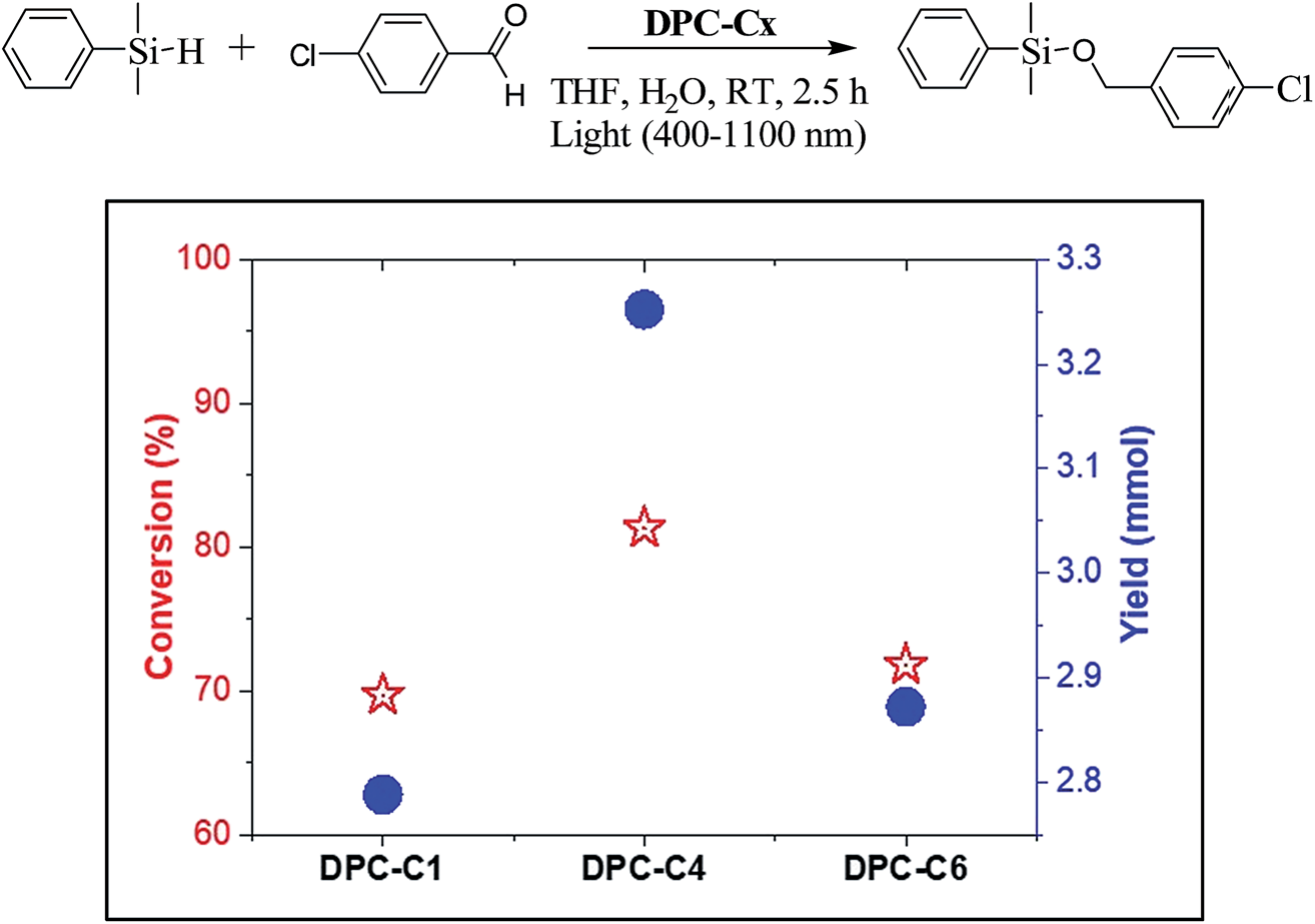
Conversion and product yield for hydrosilylation of aldehydes using DPC-C1, DPC-C4, and DPC-C6 as catalysts at 25 °C using 400–1100 nm light in 2.5 h.

To study the explicit role of “EM hotspots”, we chose the hydrosilylation of aldehydes, which does not require energetic electrons (Fig. 13). To eliminate the thermal effect, reactions were carried out at room temperature by using a water circulator. We observed a similar pattern with DPC-C4 showing the best catalytic performance for hydrosilylation of aldehydes, confirming the role of EM hot-spots on this photo-catalytic reaction also.

## 3. Conclusion

In conclusion, we have developed the solution phase synthesis of dendritic plasmonic colloidosomes with varying interparticle distances between gold NPs using a cycle-by-cycle growth approach by optimizing the nucleation–growth step. These DPCs absorbed the entire visible and near-infrared region of solar light, due to interparticle plasmonic coupling as well as the heterogeneity in the Au NP sizes, which transformed gold material to black gold. Raman thermometry and SERS provided information about the thermal and electromagnetic hotspots and local temperatures which were found to be dependent on the interparticle plasmonic coupling. The spatial distribution of the localized surface plasmon modes by STEM–EELS plasmon mapping confirmed the role of the interparticle distances in the SPR of the material.

We observed the significant effect of the plasmonic hotspots on the performance of these DPCs for the oxidation reaction of cinnamyl alcohol using pure oxygen as the oxidant, hydrosilylation of aldehydes, temperature jump assisted protein unfolding and purification of seawater to drinkable water *via* steam generation. They also catalyzed CO_2_ to methane (fuel) conversion at atmospheric pressure and temperature, using solar energy.

This was attributed to varying interparticle distances and particle sizes in these dendritic plasmonic colloidosomes. The results indicate the synergistic effects of EM and thermal hotspots as well as hot electrons on DPC-C*x* performance. Thus, DPC-C*x* catalysts can effectively be utilized as Vis-NIR light photo-catalysts, and the design of new plasmonic nanocatalysts for a wide range of other chemical reactions may be possible using the concept of plasmonic coupling.

## 4. Method

### 4.1 Synthesis of gold nanoparticles on silica spheres as seeds (DPC-C0)

Dendritic fibrous nanosilica was functionalized with (3-aminopropyl)triethoxysilane (APTES) by refluxing 4 g of calcined silica with APTES (4 mL, 17 mmol) in 250 mL of toluene at 80 °C for 24 h. The resulting material was washed repeatedly with toluene and ethanol, followed by drying under vacuum at 60 °C for 12 h. The obtained material (0.5 g) was dispersed in water (50 mL), and the mixture was sonicated for 15 min followed by 10 min of vigorous stirring at room temperature. To this reaction mixture, gold(iii) chloride hydrate salt (43 mg, taken from a stock solution of HAuCl_4_ dissolved in 10 mL water) was added dropwise. Then, the reaction mixture was sonicated for 15 min, followed by stirring for 2 h at room temperature. A freshly prepared NaBH_4_ solution (5 mL, 1 M in water) was added dropwise to this solution, which was further stirred for 2 h. The resulting material was then isolated by centrifugation, thoroughly washed with water and ethanol, and dried under vacuum at 80 °C for 12 h. The material was denoted as DPC-C0.

### 4.2 Cycle-by-cycle synthesis of dendritic plasmonic colloidosomes

DPC-C0 seeds (100 mg) were added to a previously prepared 200 mL K-gold solution (made by adding 50 mM HAuCl_4_ to a 1 mM K_2_CO_3_ solution). Then 1 mL of ammonium hydroxide (37%) was added and stirred for 5 minutes. The formaldehyde solution (18 mL) was then added dropwise to the suspension. After stirring for 24 h, the solution was centrifuged at 10 000 rpm and washed three times with deionized water and then ethanol and dried under vacuum at 80 °C for 12 h. The material was denoted as DPC-C1. These growth cycles were repeated to obtain different samples of DPC-C*x*.

### 4.3 Characterization

Scanning transmission electron microscopy (STEM) analysis was carried out on an FEI-TITAN operated at an accelerating voltage of 300 kV. For sample preparation, powders of the DPC-C*x* were dispersed in ethanol with the assistance of sonication for 10 s, and a drop of solution was dropped onto a holey carbon-coated 200 mesh TEM grid. Excess liquid was immediately wicked away with blotting paper and then air dried before exposing the sample to an electron beam and plasma cleaning for 5 s. X-ray diffraction patterns were recorded using a Panalytical X'Pert Pro powder X-ray diffractometer using Cu-Kα radiation. UV-DRS measurements were carried out using a JASCO UV/vis/NIR spectrophotometer. The surface area was obtained using the Brunauer–Emmett–Teller (BET) theory from N_2_ physisorption data recorded using a Micromeritics 3Flex analyzer. Approximately 100 mg of each sample was degassed at 120 °C for 12 h prior to N_2_ sorption analysis.

### 4.4 Water heating study

Elevation in temperature upon light illumination was studied by adding 5 mL of water and 5 mg of DPC-C*x* into a 10 mL quartz tube. The tube was wrapped with aluminum foil from the top, leaving approximately 3 cm exposed at the bottom, on which light was focused. A thermocouple was then inserted into the tube up to the level of the wrapped foil such that it was not exposed directly to incident light. The sample was then stirred at 400 rpm, and the light was focused onto the tube. The increase in temperature was measured with respect to time for 1 h with a 1 min time interval. The spectral range of the illuminating light was from 400 to 1100 nm with a power of 0.65 W cm^–2^.

### 4.5 Steam generation study

DPC-C*x* (2.5 mg) was taken up in an Eppendorf tube, and 3.5 mL of deionized water was added into it. The Eppendorf tube was vortexed for 30 s, and the solution was transferred to a quartz cuvette. The cuvette was then placed on the weighing balance and tared after stabilization of the placed cuvette. The light was then illuminated from the top of the cuvette, keeping a distance of 2 cm between the cuvette and the light guide. Weight loss, as observed from the balance, was then noted with respect to the time of light exposure. The spectral range of the illuminating light was from 400 to 1100 nm with a power of 0.69 W cm^–2^.

### 4.6 Hydrogen evolution from the ammonia borane complex

In a 50 mL Schlenk tube, 10 mg of the DPC-C*x* material was dispersed into 1 mL of water and sealed with a septum and degassed for 30 min in an argon atmosphere. The ammonia borane complex (100 μmol) was dissolved in 1 mL of water and injected into the above solution. The sample was then exposed to light with a spectral range from 400 to 1100 nm with a power of 0.55 W cm^–2^ and the evolved hydrogen was monitored using gas chromatography (GC) using a thermal conductivity detector (TCD).

### 4.7 SERS study

All samples were dispersed by sonication in ethanol. To ensure the same particle concentration, the concentration of silica (1 mg mL^–1^) was the same for each sample. The weight of DPC-C*x* needed to make a 1 mg mL^–1^ silica solution was calculated using the Au weight percent obtained from the EDS data. 50 μL of these sample solutions and 100 mM 4-aminothiophenol ethanol solution were mixed and sonicated for 30 s at room temperature. After sonication, the sample was collected by centrifuging at 8000 rpm for 5 min. The precipitate was washed with 100 μL of ethanol twice to remove the un-adsorbed chemicals. Finally, the precipitate was collected and dispersed in 25 μL of ethanol. For performing SERS measurements, a sticker chamber and slide glass were used to dry each sample separately. Raman measurements were performed using an In-*via* micro Raman setup with a 633 nm laser.

### 4.8 Raman thermometry

All samples were dispersed by sonication in ethanol. The concentration of the silica content was fixed at 1 mg mL^–1^ in all samples using Au-loading data obtained from EDS. For SERS experiments, 4-ATP is used as a Raman molecule after dissolving in ethanol. First, the same volume (50 μL) of both a sample solution and a 0.1 mM 4-ATP solution were mixed and sonicated for 30 s at room temperature. After sonication, each sample solution was dropped onto a cover glass and completely dried at room temperature. For SERS measurements, a sticker chamber and cover glass were used for drying each sample separately. All Raman spectra were acquired using an inverted microscope system (Ntegra, NT-MDT) with an objective lens (60×, air). Spectra were collected with a 0.27 mW 633 nm laser with an acquisition time of 20 s. Each spectrum was collected from different points of the dried and aggregated samples. From one point, the Stokes signal was acquired first. After changing the detection wavelength range (∼10 s), the anti-Stokes signal was collected under the same conditions.

To minimize the effects of heterogeneity in the Au coating, data from eight different points for each sample were collected. Local temperatures were estimated using the following equation (detailed calculation is given in Table S4†):^22^
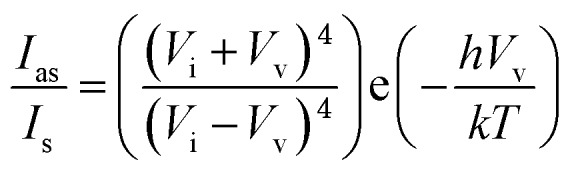
where *I*_as_ is the anti-Stokes signal intensity, *I*_s_ is the Stokes signal intensity, *V*_i_ is the laser frequency (in cm^–1^), *V*_v_ is the vibrational mode frequency (in cm^–1^), *h* is Planck's constant = 6.626176 × 10^–34^ J s, *k* is Boltzmann's constant = 1.3807 × 10^–23^ J K^–1^, and *T* is the temperature (in K).

### 4.9 EELS plasmonic mapping

The sample was mapped with EELS, and the entirety of the spectral information was gathered for each point in an image, generating a three-dimensional dataset, a spectral image. Surface plasmons were detected by placing the electron beam close to the DPC spheres and measuring the energy loss of the electrons. During the experiment, the electron beam was rastered across the silica spheres, and at each position, both the HAADF and energy-loss signals were simultaneously acquired. The resulting image was then correlated with the HAADF image to create a spatial profile. EELS spectra at different positions (1–12 in Fig. 9) were then extracted in a square window of 25 nm × 25 nm from the map. Since the surface plasmon modes lie in the region of 400 to 1100 nm (Fig. 2 and S3a†), we imaged surface plasmons in the energy range between 1.3 and 2.6 eV.

### 4.10 Catalytic study

In a 50 mL quartz tube, 10 mg of the DPC-C*x* was dispersed using 5 mL of ethanol. Cinnamyl alcohol (0.1 mmol) was then added, and the reactor was pressurized with 100 psi oxygen. It was flushed ten times with oxygen. Since the SPR of DPC-C*x* ranges from the visible to the NIR region of the spectrum, we used light radiation with a spectral width of 400–1100 nm and a light intensity of 1 W cm^–2^. Reaction progress with time was monitored using gas chromatography (GC), and the formed products were identified by mass spectrometry (MS).

For the CO_2_ conversion experiment, 10 mg of the DPC-C*x* was taken in sealed glass reactor in 5 mL isopropanol and 0.5 mL water and flushed with CO_2_ (150 mL min^–1^) for 30 minutes. The reactor was then exposed to light (400 to 1100 nm, 1 W cm^–2^) and methane formation was monitored by online micro-gas chromatography (Fig. S10†).

For hydrosilylation of aldehydes, DPC-C*x* (10 mg) was placed in a 10 mL round bottom test-tube. Tetrahydrofuran (1 mL), dimethylphenylsilane (153 μL, 1 mmol) and 4-chlorobenzaldehyde (421 μL, 3 mmol) was added and purged with argon gas. The reaction mixture was stirred at RT and the temperature of the reaction was maintained using a water circulator. The reactor was then exposed to light (400 to 1100 nm) and product formation was monitored by gas chromatography-mass spectroscopy.

## Conflicts of interest

There are no conflicts to declare.

## Supplementary Material

Supplementary information

Supplementary movie

Supplementary movie

Supplementary movie

Infographic
