# Supplementary material for: Plasmonic colloidosomes of black gold for solar energy harvesting and hotspots directed catalysis for CO2 to fuel conversion
Source: Chem Sci. 2019 Jul 3;10(27):6594–603. doi: 10.1039/c9sc02369k (PMC6625417; doi:10.1039/c9sc02369k)
Supplement: Supplementary file 1 [file SC-010-C9SC02369K-s001.pdf]

Electronic Supplementary Material (ESI) for Chemical Science.
This journal is © The Royal Society of Chemistry 2019

Plasmonic Colloidosomes of Black Gold for Solar Energy Harvesting and Hotspots Directed Catalysis for CO_2 to Fuel Conversion

Mahak Dhiman,[a]# Ayan Maity,[a]# Anirban Das,[a] Rajesh Belgamwar,[a] Bhagyashree Chalke,[b] Yeonhee Lee,[c] Kyunjong Sim,[c] Jwa-Min Nam,[c] Vivek Polshettiwar*[a]

[a]Department of Chemical Sciences, [b]Department of Condensed Matter Physics and Materials Science, Tata Institute of Fundamental Research (TIFR), Mumbai, India. Email: vivekpol@tifr.res.in,

[c]Department of Chemistry, Seoul National University, Seoul, South Korea. # shared co-authors

Table S1. Au Loading, BET surface area and total pore volume of DPC-Cx with various growth cycles.

Entry	Sample	Au loading (wt. %)	BET Surface Area (m^2/g)	Total BJH Pore Volume (cm^3/g)
1	Silica Spheres	0	551	0.6
2	DPC-C0	4.9	359	0.5
3	DPC-C1	22	340	0.4
4	DPC-C3	48	347	0.5
5	DPC-C4	55	244	0.4
6	DPC-C6	68	134	0.2

Standard error in measurements: Au loading ± 10 %, surface area ± 4%, pore volume ± 0.01.

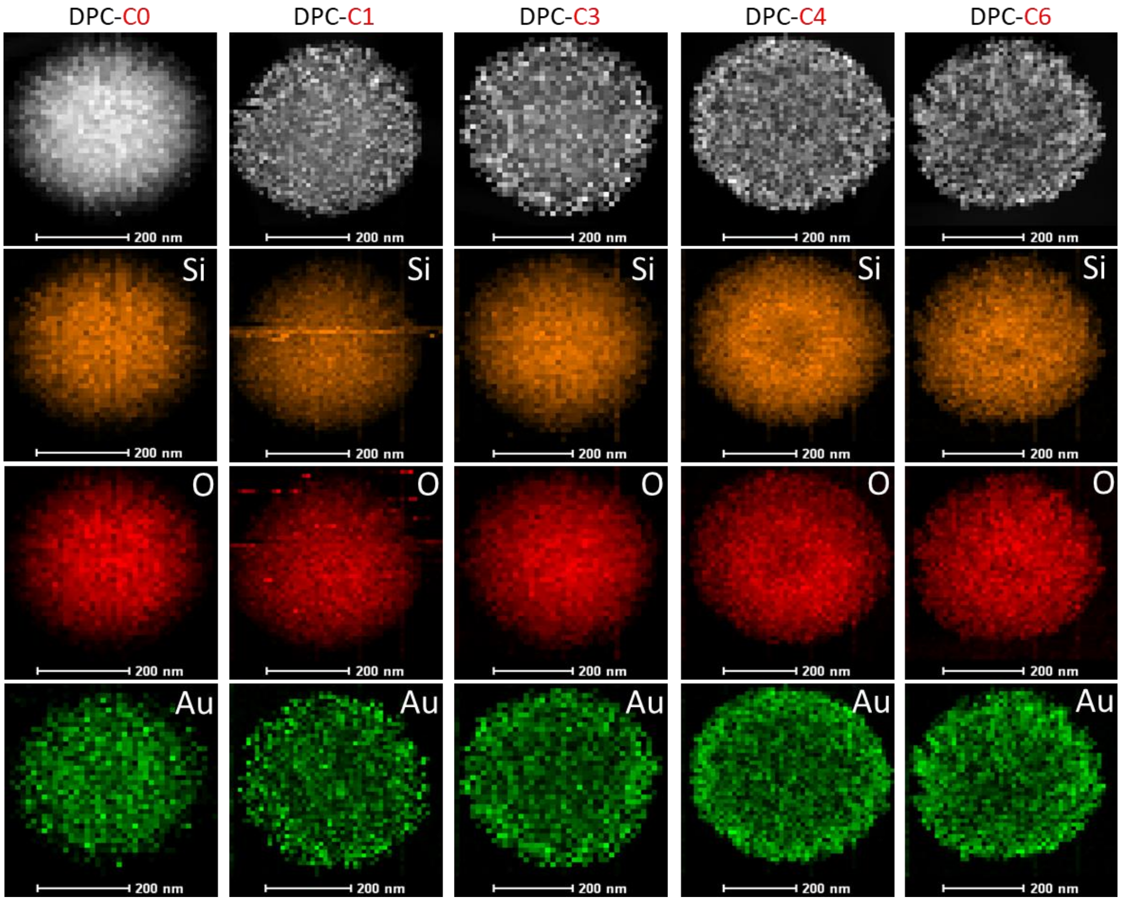

Figure S1. EDS elemental mapping of DPC-Cx for Si, O and Au atoms.

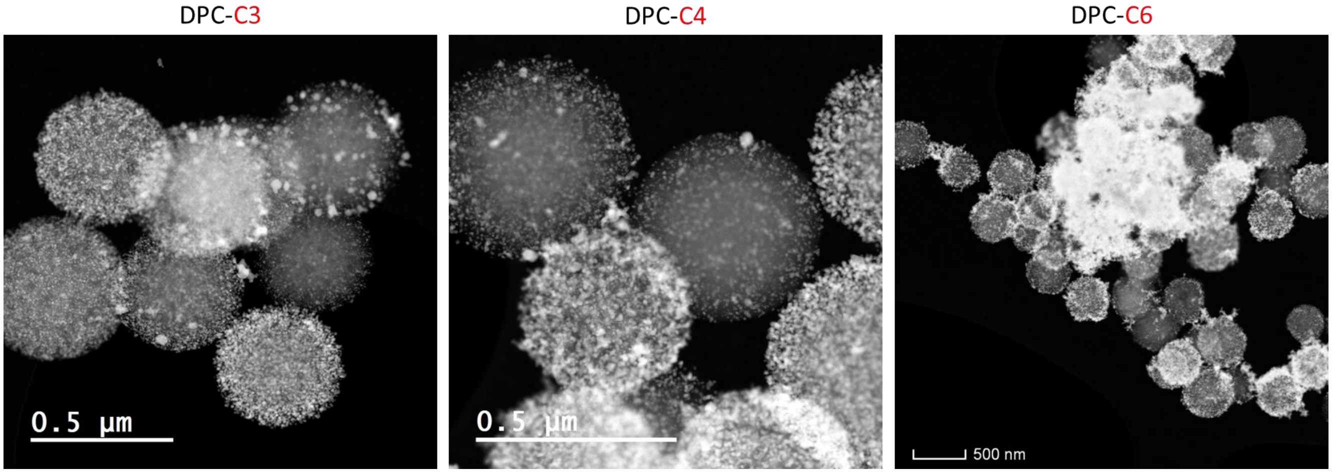

Figure S2. Low magnification STEM image of DPC-C3, DPC-C4 and DPC-C6.

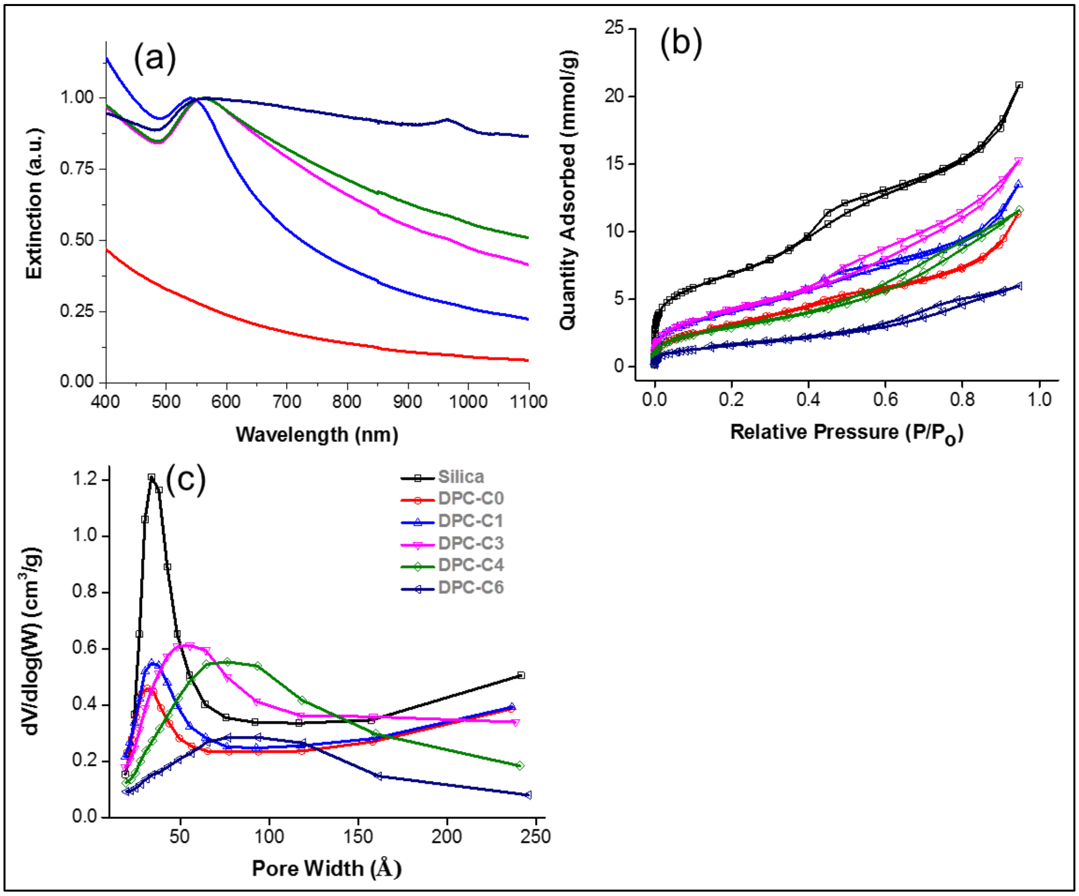

Figure S3. (a) Extinction spectrum, (b) N_2 sorption isotherms and (c) BJH pore size distribution of DPC-Cx with various numbers of growth cycles.

All N_2 sorption isotherms were of type IV with a H3 hysteresis loop. The pore-size distribution curves (Figure S3c) indicate that the pores, which were approximately ~ 4 nm, were blocked after amine functionalization, while the mesopores remained intact even after Au loading. This allowed the better accessibility of Au NPs, as well as enhanced mass transport of reactants and products. The surface area and pore volume of DPC-Cx are summarized in Table S1. In terms of porosity, despite high Au loading, DPC-Cx materials possessed considerable porosity (Figure S3b). Surface area was reduced from 551 m^2g^{-1} of silica spheres to 340 m^2g^{-1} of DPC-C1 after 1st growth cycle due to 22 wt.% Au loading (Table S1). The surface area was further decreased in DPC-C3, DPC-C4 and DPC-C6 and was measured to be 347, 244 and 134 m^2g^{-1}, respectively (Table S1).

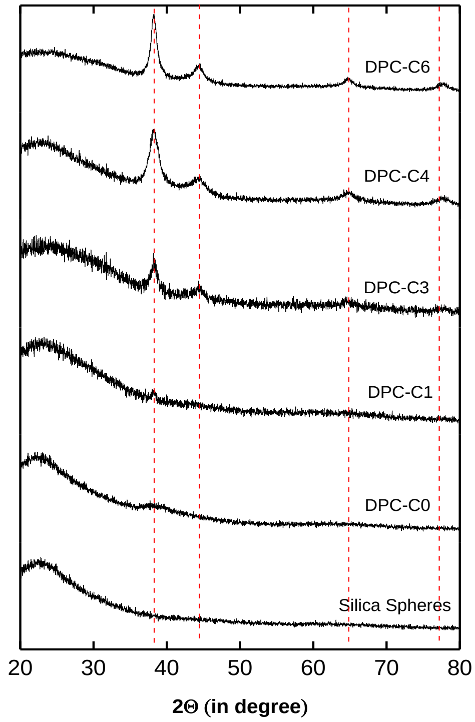

Figure S4. Powder X-ray diffraction (PXRD) of DPC-Cx with various growth cycles.

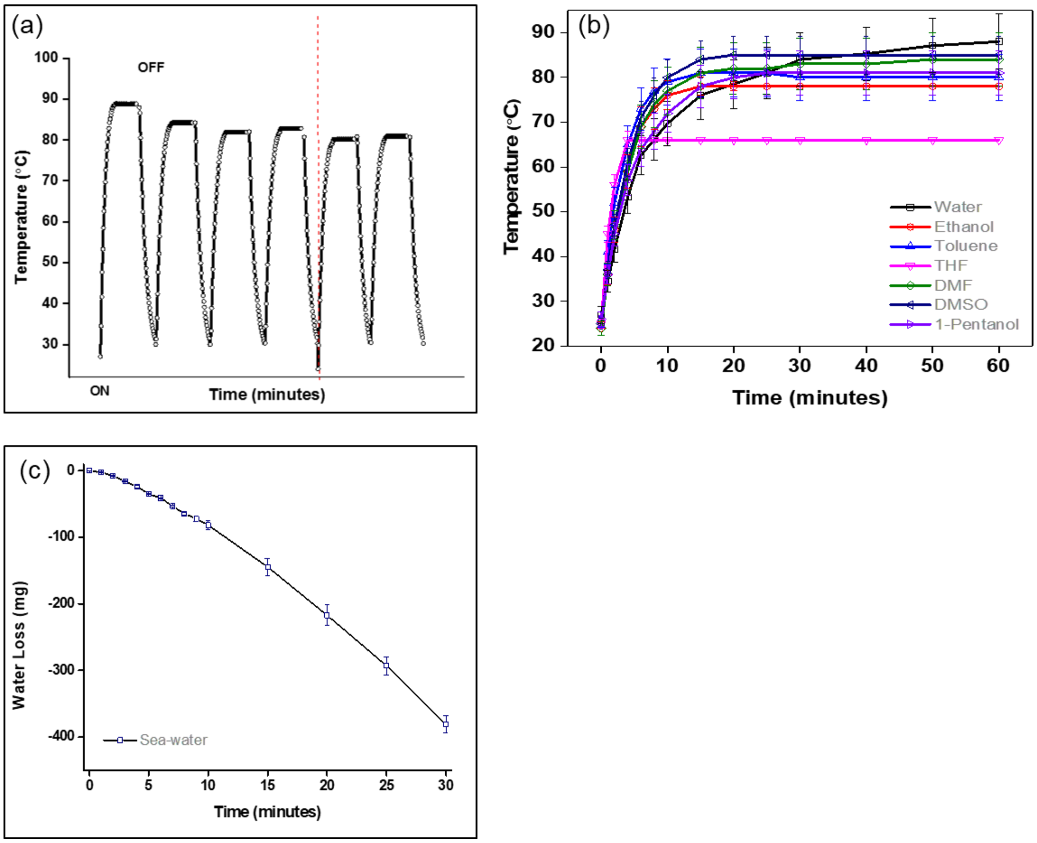

Figure S5. (a) DPC-C4 recyclability for water heating experiments for six heating-cooling cycles. To check the stability of the DPC-C4 during the heat generation, multiple cycles of heat measurements were carried out by switching light ON and OFF for 30 minutes of the time interval. The experiment was performed by exposing 5 mg of DPC-C4 catalyst in 5 mL of water to Vis-NIR light (400-1100 nm) for 30 minutes and then switching off the light for the next 30 minutes. Once the temperature reached 30°C, the light was again switched ON and the increase in temperature with respect to time was monitored. Red line indicates the gap of 24 hours before resuming further recyclability study; (b) Effect of solvent on DPC-C4 heating behavior; (c) DPC-C4 as nano-heaters to convert seawater to drinkable water.

Table S2. Measurement of thermal efficiency of the steam generation process.

Thermal efficiency (η) of the photothermal material was estimated by the following equation:

$$\eta = \frac{Qe}{Qs}$$

Where Qs is the incidence of light power, and Qe is the power of evaporation of the water, which can be estimated by the equation:

$$Qe = \frac{dm * He}{dt} = v * He$$

Where m is the mass of evaporated water,

t is time,

v is the evaporation rate of water, and

He is the heat of evaporation of water (≈2260 kJ kg $^{-1}$).

**Sample**	v **(kg m^{-2} h^{-1})**	Qe **(kJ m^{-2} h^{-1})**	Qs **(W m^{-2})**	**η (%)**
DPC-C0	2.6	5966	6900	24
DPC-C1	3.5	7910	6900	32
DPC-C3	7.2	16272	6900	65
DPC-C4	8.6	19436	6900	78
DPC-C6	7.1	16046	6900	64

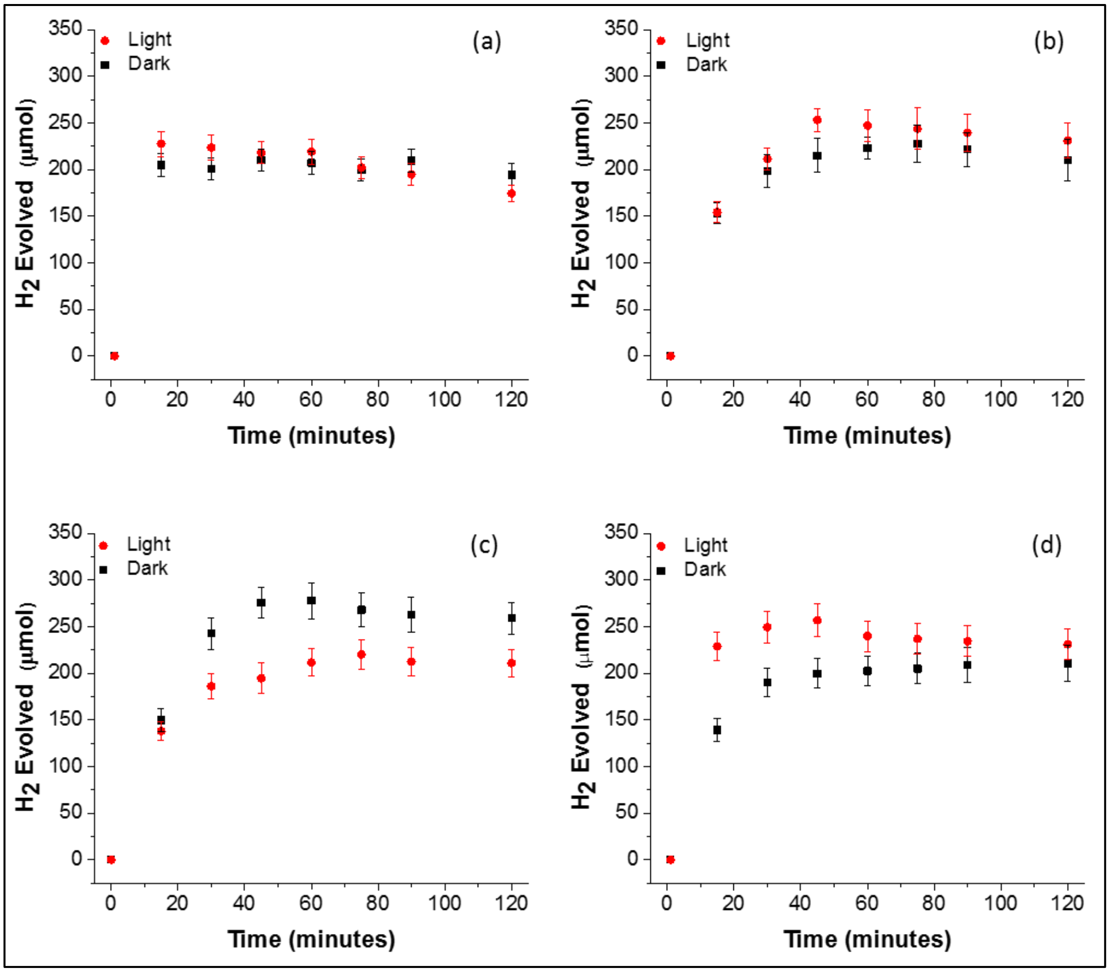

Figure S6. ABC decomposition in the dark at respective temperatures and in light, (a) using DPC-C1 at 64°C, (b) using DPC-C3 at 70°C, (c) using DPC-C4 at 74°C and (d) using DPC-C6 at 68°C.

Table S3. Measurement of localized temperatures in DPC-Cx materials.

Temperature was determined using the following equation:

$$\frac{Ias}{Is} = \left(\frac{(Vi + Vv)^4}{(Vi - Vv)^4}\right) e\left(-\frac{hVv}{kT}\right)$$

where;

Ias is the anti-Stokes signal strength,

Is is the Stokes signal strength,

Vi is the laser frequency (15797.8 cm^{-1}),

Vv is the vibrational mode frequency (in cm^{-1}),

h is Planck's constant = 6.626176 x 10^{-34} Js,

k is Boltzmann's constant = 1.3807 x 10 $^{-23}$ JK^{-1}, and

T is the temperature (in K).

To minimize the effect of heterogeneity in Au-coating on temperature, data was acquired from eight different points for each sample.

The temperature was determined using the following steps:

1. The ratio of *Ias* and *Is* was taken, $(\frac{Ias}{Is})$
2. To simplify the final equation for temperature calculation, a factor Z was introduced where,

$$Z = (\frac{Ias}{Is}) \;/\; \left(\frac{(Vi+Vv)^4}{(Vi-Vv)^4}\right)$$

3. Finally, the temperature (T) was determined as,

$$T = -\frac{hVv}{(k\,\mathrm{Ln}(Z))}$$

Sample	Point	Ias	Is	Ias/Is	Vv	Z	Temp. (K)	Temp. (^oC)
**DPC-C3**								
	1	22	1315	0.01673	1071	0.009718	333	60
	2	82	2354	0.034834	1070	0.020245	395	122
	3	68	2629	0.025865	1070	0.015033	367	94
	4	71	1518	0.046772	1071	0.02717	428	155
	5	70	1931	0.036251	1071	0.021058	399	126
	6	98	2366	0.04142	1070	0.024073	413	140

	7	43	1174	0.036627	1071	0.021276	400	127
	8	118	3294	0.035823	1068	0.020841	397	124
**DPC-C4**								
	1	145	3089	0.046941	1068	0.027309	512	154
	2	38	1307	0.029074	1073	0.016872	443	105
	3	101	2403	0.042031	1070	0.024428	494	142
	4	142	4805	0.029553	1068	0.017193	443	105
	5	120	3108	0.03861	1070	0.02244	481	133
	6	74	2009	0.036834	1071	0.021397	475	128
	7	30	850	0.035294	1073	0.020481	469	124
	8	67	2838	0.023608	1068	0.013735	416	86
**DPC-C6**								
	1	53	2863	0.018512	1073	0.010743	341	68
	2	205	7003	0.029273	1066	0.017048	377	104
	3	161	5963	0.027	1071	0.015684	371	98
	4	73	2404	0.030366	1071	0.017639	382	109
	5	146	4581	0.031871	1070	0.018523	386	113
	6	204	5081	0.04015	1066	0.023382	409	136
	7	85	2787	0.030499	1075	0.017681	384	111
	8	151	5236	0.028839	1073	0.016735	378	105

** There was a minor shift in Stokes & anti-Stokes lines for C-S stretching mode of ± 5 cm^{-1}. Raman spectrum was mixed with Raman reporter signal and background signal. So, the background subtraction process was applied.*

Table S4: SERS signals of 4-ATP on DPC-Cx materials.

SERS Peak (cm^{-1})	Peak Assignments
1005	γ (CC) + γ (CCC)
1073	υ (CS)
1137	υ (CH)
1182	δ (CH)
1385	υ (NC)
1429	υ (CC) + δ (CH)
1570	υ (CC)

Table S5. Catalysis optimization for the cinnamyl alcohol (COL) oxidation reaction. Selectivity reported was for cinnamaldehyde (CAL).

Catalyst Name	Catalyst Amount (mg)	Light/ Dark	Temperature (^{o}C)	COL (mmol)	Conversion (%)	Selectivity (%)
DPC-C1	20	Light	23	0.5	20	96
DPC-C1	20	Dark	23	0.5	9	97
DPC-C4	20	Dark	68	0.5	11	97
DPC-C4	10	Light	23	0.5	30	94
DPC-C4	10	Light	23	0.1	90	78
DPC-C4	5	Light	23	0.1	12	99
DPC-C4	10	Dark	23	0.1	7	99
DPC-C4	10	Dark	78	0.1	24	94

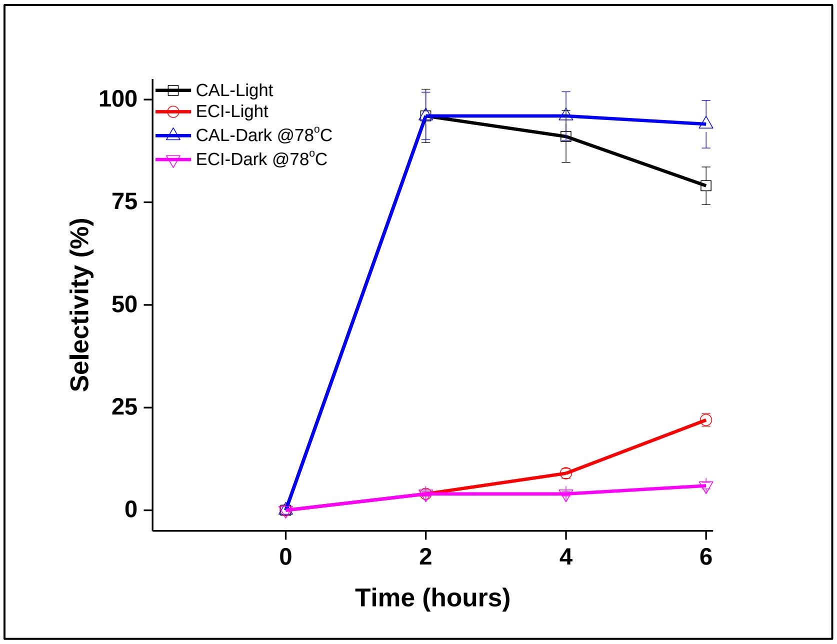

Figure S7. The difference in selectivity between cinnamaldehyde (CAL) and ethyl cinnamate (ECI) in light and dark at respective temperature, using DPC-C4 as a catalyst.

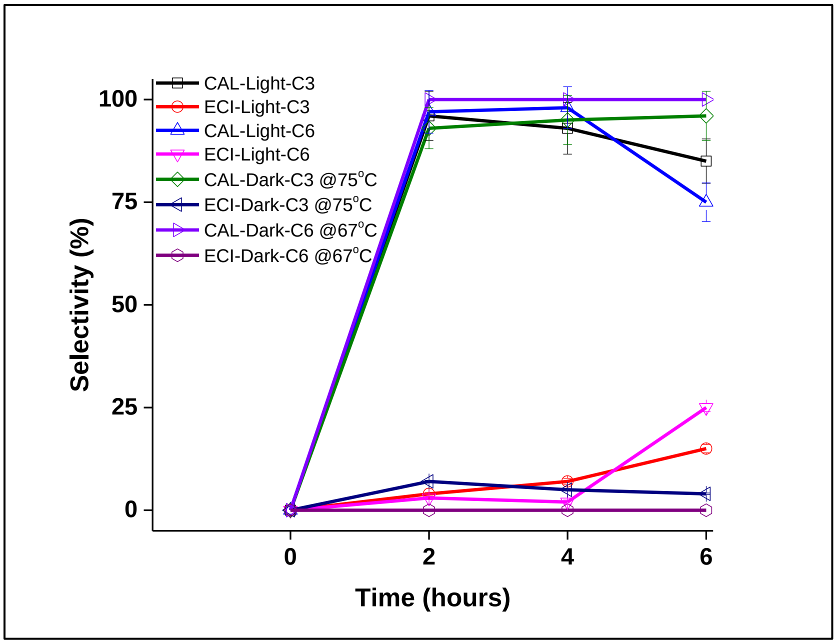

Figure S8. The difference in selectivity between cinnamaldehyde (CAL) and ethyl cinnamate (ECI) in light and dark at respective temperature, using DPC-C3 and DPC-C6 as catalysts.

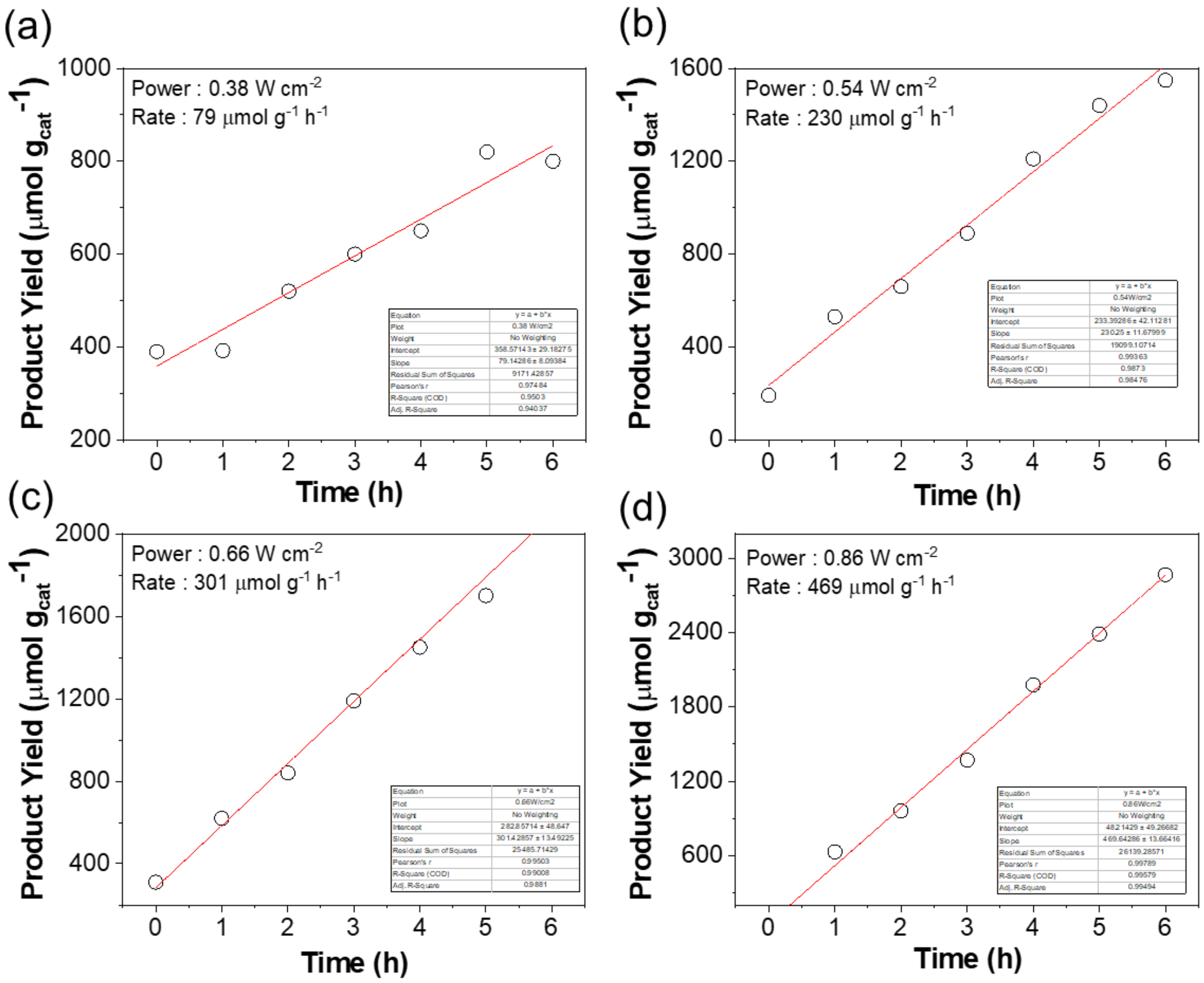

Figure S9. Product yield for hydrosilylation of aldehydes using DPC-C4 using VIS-IR light with varying intensities, a) 0.38, b) 0.54, c) 0.66, and d) 0.86 W cm^{-2}.

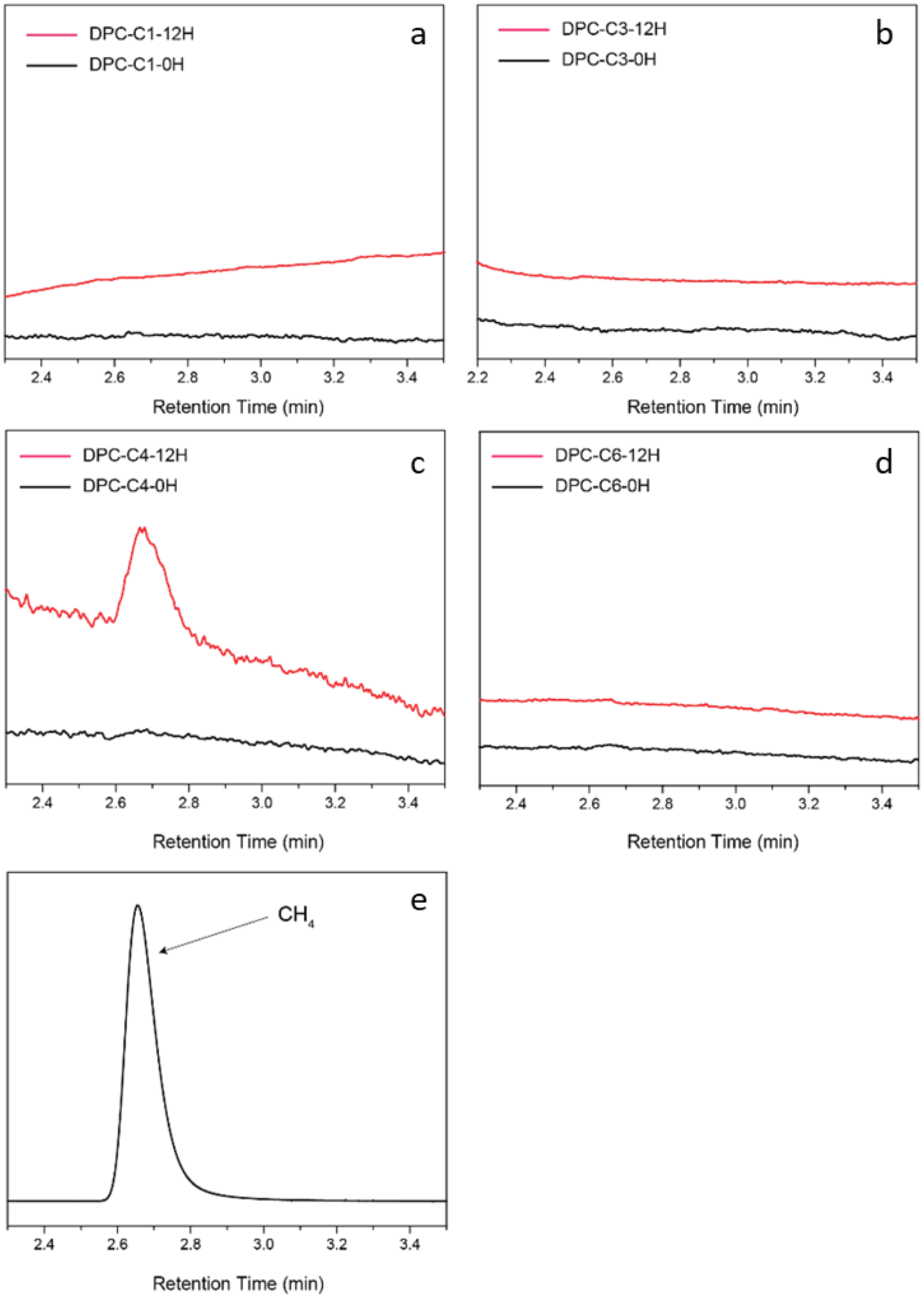

Figure S10. Detection of the product (methane) of photocatalytic CO_2 reduction reaction catalyzed by a) DPC-C1, (b) DPC-C3, (c) DPC-C4 and (d) DPC-C6, using micro-GC with thermal conductivity detector (TCD); (e) methane signal retention time of pure methane gas.
