# Supplementary material for: Plasmonic colloidosomes of black gold for solar energy harvesting and hotspots directed catalysis for CO2 to fuel conversion
Source: Chem Sci. 2019 Jul 3;10(27):6594–603. doi: 10.1039/c9sc02369k (PMC6625417; doi:10.1039/c9sc02369k)

Electronic Supplementary Material (ESI) for Chemical Science.
This journal is © The Royal Society of Chemistry 2019

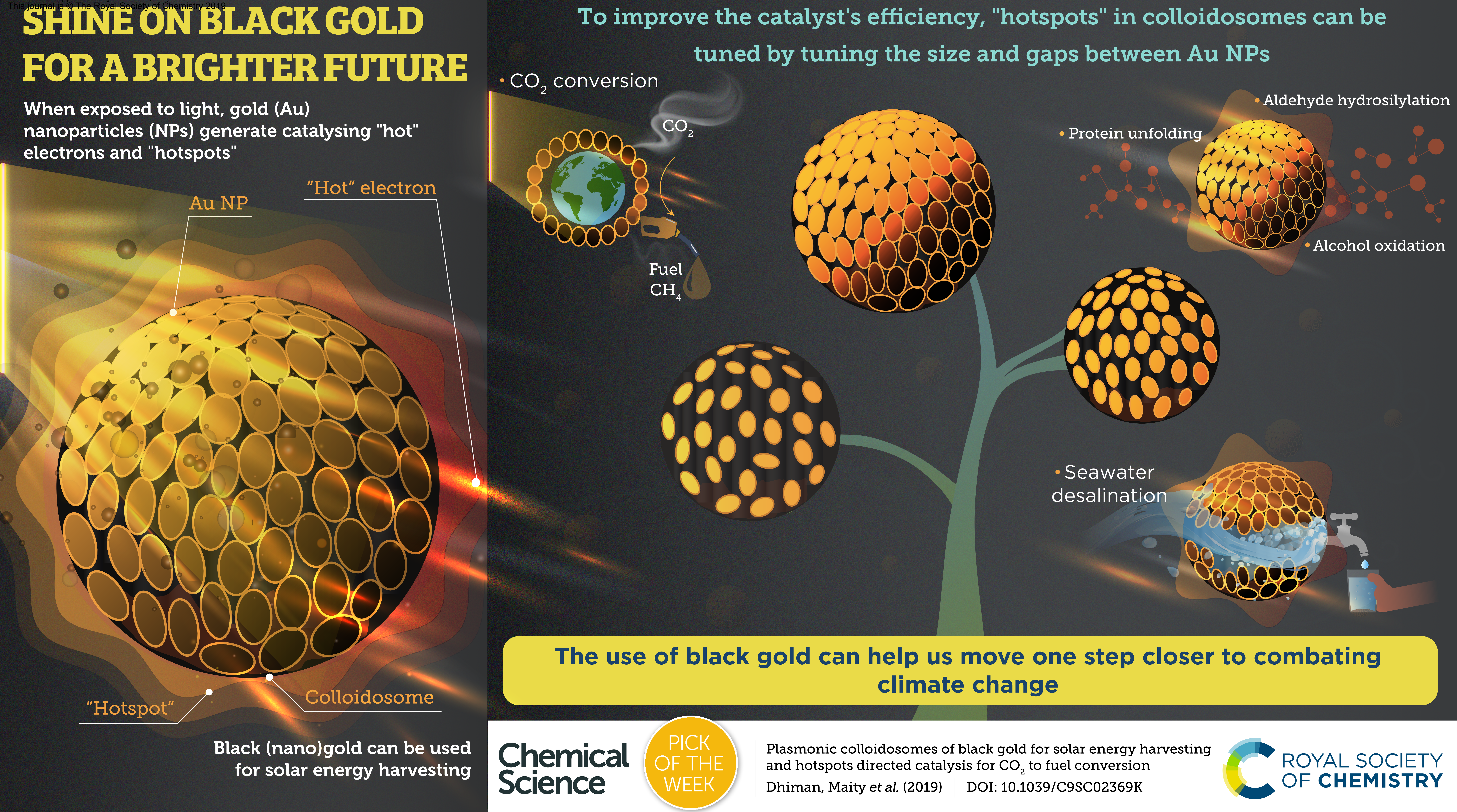

Supplement: Supplementary file 5 [file SC-010-C9SC02369K-s005.pdf]
